# Comparative analysis of single and combined APP/APLP knockouts reveals reduced spine density in APP-KO mice that is prevented by APPsα expression

**DOI:** 10.1186/2051-5960-2-36

**Published:** 2014-03-31

**Authors:** Sascha W Weyer, Marta Zagrebelsky, Ulrike Herrmann, Meike Hick, Lennard Ganss, Julia Gobbert, Morna Gruber, Christine Altmann, Martin Korte, Thomas Deller, Ulrike C Müller

**Affiliations:** 1 Department of Bioinformatics and Functional Genomics, Ruprecht-Karls University Heidelberg, Institute of Pharmacy and Molecular Biotechnology, Im Neuenheimer Feld 364, Heidelberg D-69120, Germany; 2 Present address: Department of Applied Tumor Biology, Ruprecht-Karls University Heidelberg, Institute of Pathology, University of Heidelberg, Heidelberg D-69120, Germany; 3 TU Braunschweig, Zoological Institute, Cellular Neurobiology, Spielmannstr. 7, Braunschweig D-38106, Germany; 4 Goethe University Frankfurt, Institute of Clinical Neuroanatomy, Neuroscience Center, Theodor-Stern-Kai 7, Frankfurt am Main D-60596, Germany

**Keywords:** Alzheimer, Amyloid precursor protein, Neuronal morphology, Spine density, Knockout

## Abstract

Synaptic dysfunction and synapse loss are key features of Alzheimer’s pathogenesis. Previously, we showed an essential function of APP and APLP2 for synaptic plasticity, learning and memory. Here, we used organotypic hippocampal cultures to investigate the specific role(s) of APP family members and their fragments for dendritic complexity and spine formation of principal neurons within the hippocampus. Whereas CA1 neurons from APLP1-KO or APLP2-KO mice showed normal neuronal morphology and spine density, APP-KO mice revealed a highly reduced dendritic complexity in mid-apical dendrites. Despite unaltered morphology of APLP2-KO neurons, combined APP/APLP2-DKO mutants showed an additional branching defect in proximal apical dendrites, indicating redundancy and a combined function of APP and APLP2 for dendritic architecture. Remarkably, APP-KO neurons showed a pronounced decrease in spine density and reductions in the number of mushroom spines. No further decrease in spine density, however, was detectable in APP/APLP2-DKO mice. Mechanistically, using APPsα-KI mice lacking transmembrane APP and expressing solely the secreted APPsα fragment we demonstrate that APPsα expression alone is sufficient to prevent the defects in spine density observed in APP-KO mice. Collectively, these studies reveal a combined role of APP and APLP2 for dendritic architecture and a unique function of secreted APPs for spine density.

## Introduction

A hallmark of Alzheimer´s disease (AD) is the deposition of Aβ peptides, produced by sequential cleavage of the amyloid precursor protein (APP) by β- and γ-secretase. In the competing non-amyloidogenic pathway that is meditated by α-secretase, APP cleavage occurs at a membrane-proximal site within the Aβ region. Thus, α-secretase processing not only precludes the formation of Aβ peptides but also liberates the large soluble, neuroprotective ectodomain APPsα that is secreted into the extracellular space [1-3]. Classically, synapse loss in AD has mainly been attributed to the synaptotoxic effects of various Aβ species. However, there is increasing evidence that reduced levels of APPsα and loss of APP-mediated functions may contribute to cognitive dysfunction in AD and augmentation of α-secretase activity has been suggested as a therapeutic approach for AD [4,5]. APP is a member of a small gene family that includes in mammals the APP-like proteins APLP1 and APLP2 (reviewed in [6]). Although APLPs lack the Aβ region, they share with APP several highly conserved domains, are similarly processed by the same secretases and serve partially redundant functions. Although the physiological functions of APP and APLPs are not fully understood, evidence from mouse models indicates a key role of the APP family for neuronal migration, synaptogenesis, synaptic function and plasticity [6-8]. APP knockout (KO) mice showed reduced grip strength, agenesis of the corpus callosum and impaired spatial learning associated with deficient long-term potentiation (LTP) [9-15]. Interestingly, we could previously demonstrate that APPsα serves a key functional role, as APPsα knockin (APPsα−KI) mice expressing only secreted APPsα rescued the deficits of APP-KO mice, including impairments in grip strength, spatial learning and synaptic plasticity [15].

At the cellular level, the role of APP and its proteolytic fragments for neuronal morphology appears complex. Transgenic APP overexpression decreased spine density in aged animals, whereas prior to the onset of plaque deposition increases in spine density were observed (reviewed in [16]). Conversely, age APP-KO mice showed alterations in dendritic arborization [14] and reduced spine densities on dendrites of cortical and hippocampal neurons [17]. In contrast, earlier studies found an increase in spine density in the cortex of young APP-KO mice [18] and in autaptic hippocampal APP-KO cultures [19]. These conflicting results might be related to the neuronal cell type and/or age of animals studied, or may be confounded by APLP mediated redundant functions. Indeed, mice lacking single family members are viable, whereas combined APP^-/-^APLP2^-/-^ and APLP1^-/-^APLP2^-/-^ mice die shortly after birth due to impaired neuromuscular transmission [20,21].

Nonetheless, the specific versus redundant function(s) of APP family members for neuronal architecture and spine density and the role of their various proteolytic fragments remained unclear. Here, we performed a systematic comparative analysis of neuronal morphology in the hippocampus of single and combined APP/APLP2-KO mice. These studies revealed a combined and partially redundant role of APP and APLP2 for dendritic architecture and a unique function of APP and secreted APPsα for spine density.

## Material and methods

### Preparation of slice cultures

Organotypic hippocampal slice cultures were prepared as previously described [22]. P0 mice were decapitated, the hippocampi were dissected in ice-cold Gey’s Balanced Salt Solution (GBSS, containing: kynurenic acid (0.5 μM), adjusted to pH 7.2), sliced transversely at a thickness of 400 μm using a tissue chopper (McIllwain, Wood Dale, IL) and kept for 30 min at 4°C in GBSS. The slices were plated onto Millicell-CM membrane inserts (Millipore, Bedford, MA) and cultivated (37°C, 7% CO_2_). Three days after preparation, a mixture of anti-mitotic drugs (uridine, cytosine-β-D-arabinofuranoside hydrochloride, 5-fluoro-2’-deoxyuridine) was applied for 24 h in order to reduce the number of non-neuronal cells. In addition, entorhino-hippocampal slice cultures (see [23], with minor modifications) of Thy1-GFP × APP-KO mice (P3-5) were prepared and cultivated for 12-14 days prior to imaging (age at analysis was thus equivalent to cultures prepared at P0).

### Particle-mediated transfection

Organotypic hippocampal cultures (OHCs) were biolistically transfected at DIV14 using the Helios Gene Gun system (Bio-Rad) including a modified gene gun barrel (for details see [24]). Plasmid coated microcarriers (gold, diameter 0.6 μm) were shot onto the slices using a helium burst of 70 PSI. To avoid tissue damage by larger particles, culture inserts with a pore width of 3 μm were used as filters. Bullets for transfection were prepared according to the manufacturer’s instructions (BioRad). Briefly, 2 μg plasmid DNA per mg gold was coated onto 0.6 μm gold particles. A membrane targeted farnesylated EGFP (fEGFP) under control of the neuron specific synapsin promoter was transfected, allowing the visualization of the complete dendritic tree. DNA precipitation (CaCl_2_) and coating onto gold microcarriers was performed following the protocol described by [25]. OHCs were fixed with PFA (4%) three days post-transfection.

### Neuronal imaging and analysis

The gene gun mediated delivery of a single gold microcarrier carrying the fEGFP expression plasmid results in the intense labeling of the whole dendritic tree as well as all dendritic spines of an individual pyramidal neuron. For the analysis we have selected only non-overlapping labeled neurons to obtain an unambiguous reconstruction of the entire dendritic arborization. The CA1 neurons selected for analysis were imaged with a Nikon A1R confocal microscope using a 488 nm laser. Each neuron was first imaged using a 20 × objective (Numerical aperture (NA): 0.8) and z-sectioned at 1 μm increments to obtain a three-dimensional reconstruction of the entire dendritic tree, subsequently used for Sholl analysis of total dendritic length and complexity [26]. The morphometric Sholl analysis was obtained from reconstructed neurons using the Neurolucida and NeuroExplorer software (MicroBrightField, Williston, USA). In short, a series of concentric spheres (centered around the soma) were drawn with an intersection interval of 30 μm and the number of dendrites crossing each sphere was calculated. This analysis was done separately for basal and apical dendrites and was plotted against the distance from the soma. We also calculated the total dendritic complexity, which reflects the total number of dendrite crossings for each pyramidal neuron using 10 μm increments. For analysis of spine density in OHCs identical parts of basal and mid-apical dendrites were imaged at a higher magnification with a 60 × 1.4 NA Plan-APO oil immersion objective (zoom factor 1.8) and a z-stack thickness of 0.15 μm. Deconvolution of confocal stacks was done using Huygens Deconvolution Analysis software (Nikon Imaging Center, Heidelberg University). Only protrusions emanating laterally from the dendrites and exceeding the threshold of 0.3 μm were included for spine analysis (similar to [27], with minor alterations). All neurons were imaged and analyzed blind to genotype. In entorhino-hippocampal slice cultures GFP-labeled CA1 neurons were imaged using an upright Zeiss confocal microscope. Quantitative analysis was performed in line with Holtmaat et al. [27]. Only lateral spines were counted exceeding a threshold of 0.4 μm from the dendrite. Again, neurons were analyzed blind to genotype.

### Classification of spines

Dendritic spines were classified as stubby-, mushroom- and thin- shaped spines [28] according to their length (from the base up to the tip of their head) and the ratio between the minimum neck (min_neck_) and the maximum spine head (max_head_) diameter [29-31]. The following criteria were used for spine subtype classification: stubby (length < 1 μm, max_head_/min_neck_ ratio < 1.5), mushroom (max_head_/min_neck_ ratio ≥ 1.5, independent of spine length), thin (length ≥ 1 μm, max_head_/min_neck_ ratio < 1.5).

### Statistical analysis

Values obtained for spine density, dendrite number or dendritic length were exported to Microsoft Excel or Graphpad Prism for statistical analysis. Comparison of group means was performed using Student’s t-test or ANOVA with an appropriate post hoc test as stated in the results section. Sholl analysis data were compared using repeated measure ANOVA with genotype as the between group factor and distance from soma as the repeated-measure factor. Bonferroni multiple comparison test was used to compare means of a defined distance to soma between different genotypes. All data shown are presented as mean ± SEM if not stated otherwise. Level of significance was set at p < 0.05.

### Electrophysiological recordings in organotypic hippocampal cultures

After placing the OHCs in a submerged recording chamber, field excitatory postsynaptic potentials (fEPSPs) were recorded in the stratum radiatum of the CA1 region with a glass micropipette (resistance 2-10 MΩ) filled with 3 M NaCl at a depth of ~100-150 μm. Investigation of the basal synaptic transmission was performed by correlating fEPSP sizes to defined stimulus intensities (input-output curve, 8-22 μA in steps of 2 μA). Presynaptic function and short-term plasticity were explored by using paired-pulse facilitation (PPF) paradigm with interstimulus intervals (ISI) ranging from 10, 20, 40, 80 to 160 ms. Data were collected, stored and analyzed with LABVIEW software (National Instruments, Austin, TX). The initial slope of fEPSPs elicited by stimulation of the Schaffer collaterals was measured over time, and plotted as average ± SEM.

### Mice

The generation and genotyping of knockout lines was formerly described: APP-KO and APLP1-KO [20]; APLP2-KO [32]; APPsα-KI [15]. APP/APLP2 double knockout mice (DKO) were generated by three consecutive crosses. In short, APP-KO single mutants were intercrossed with APLP2-KO mice to obtain mice heterozygous for both loci. After backcrossing these mice onto an APLP2-KO background, heterozygous mice (APP^+/-^/APLP2^-/-^) were intercrossed to generate APP/APLP2-DKO mice and APLP2-KO littermate controls. All mice have been backcrossed at least six times to C57BL/6 mice that were used as WT controls. All mice were bred and housed under identical conditions in the same room throughout their lifetime. Crossbreeding of APP-KO with Thy1-GFP mice [33] yielded Thy1-GFP expressing APP-KO and APP-WT mice. From pups of these lines, entorhino-hippocampal slice cultures were prepared.

### Western blot analysis

The fractionation of soluble APPsα and cell bound holoAPP was performed as described by [15]. Briefly, to preserve cell membranes, brain hemispheres were homogenized in 10 volumes of sucrose containing tissue homogenization buffer (THB: 250 mM sucrose, 20 mM Tris-HCl pH 7.4, 1 mM EDTA, 1 mM EGTA, protease inhibitor cocktail) using a Potter-S Homogenisator (B. Braun). Remaining tissue fragments were removed by a low speed spin (5.000 × g, 5 min.). This 10% sucrose homogenate was mixed with an equal volume of 0.4% diethylamin (DEA), 100 mM NaCl and homogenized again by pottering. After high-speed centrifugation (100.000 × g, 1 h, 4°C) the APPsα containing supernatant (SN) was recovered, neutralized with 0.5 M Tris base pH 6.8 and mixed with 2× sample buffer for loading. Gel loading was normalized to total protein content determined by the BCA method (Pierce). Samples were separated by SDS-PAGE (10% Tris/tricine gels). Proteins were electroblotted onto polyvinylidene fluoride membrane (Immobilon-P, Millipore) and detected by Western blotting using monoclonal m3.2 antibody (at 1:1000), specific for the C-terminus of APPsα [34]. Antibody directed against β-tubulin was from Millipore (MAB3408, 1:10.000).

The study complies with German legislation on animal welfare and experimentation.

## Results

### CA1 pyramidal cells of APLP2-KO and APLP1-KO mice show normal neuronal morphology

**Figure 1:**
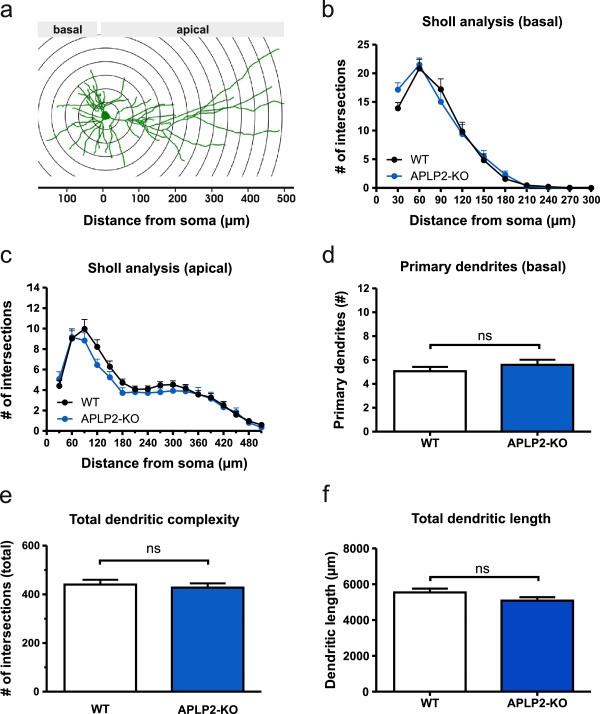
**APLP2-KO CA1 neurons show a WT-like morphology. (a)** Schematic representation of morphometric Sholl analysis. The number of intersections between dendrites and concentric spheres centered on the soma was determined at various distances from soma (30 μm increments). Sholl analysis of basal **(b)** and apical dendrites **(c)** of CA1 pyramidal neurons from APLP2-KO and WT mice revealed no significant alterations in dendritic morphology (Repeated measures ANOVA with Bonferroni’s multiple comparison test, n.s.). Neuron reconstruction and analysis were done using the Neurolucida and Neuroexplorer software (Microbrightfield). **(d-f)** Neither significant alterations in the number of primary basal dendrites (WT: 5.06 ± 0.37 versus APLP2-KO: 5.60 ± 0.43; Student’s t-test p > 0.05) **(d)** nor changes in the total dendritic complexity **(e)** or total dendritic length **(f)** were observed (Student´s t-test, n.s.). WT: n = 32 neurons/ 6 mice, APLP2-KO: n = 25 neurons/ 5 mice. Values represent mean ± SEM.

We used single or combined KO mice to investigate the role of APP family members for neuronal morphology. As a combined APP/APLP2 double knockout (DKO) is lethal early after birth we analyzed morphology of pyramidal cells in organotypic hippocampal cultures (OHCs) prepared at postnatal day 0 (P0). Cultures were biolistically transfected with membrane-targeted farnesylated EGFP (fEGFP) which resulted in a small random population of fEGFP expressing neurons that were imaged and digitally reconstructed at day *in vitro* 17 (DIV17). We focused on CA1 as this region is highly vulnerable in AD, is one of the best studied brain regions with regard to synaptic plasticity, and we had previously demonstrated LTP defects at CA3/CA1 synapses in APP/APLP mutant mice [15,35]. To this end, we first studied APLP2-KO cultures as compared to wild type (WT) cultures. No apparent alterations in dendritic orientation or gross neuronal architecture were observed when qualitatively comparing reconstructed mature CA1 neurons of APLP2-KO mice with WT neurons. In view of their different morphology and connectivity we analyzed apical and basal dendrites of CA1 neurons separately. Performing morphometric Sholl analysis we plotted the number of intersections with circles centered on the soma against the distance from the cell body (Figure 1a-c). For the measurement of dendritic complexity (Figure 1e) the complete dendritic arbor was analyzed. Detailed Sholl analysis of APLP2-KO neurons revealed unaltered complexity for both basal (Figure 1b) and apical (Figure 1c) dendrites (Repeated measures ANOVA; basal: Genotype F_(1,55)_ = 0.08081, p = 0.78, ns; apical: Genotype F_(1,55)_ = 1,551, p = 0.22, n.s.).

Consistent with Sholl analysis neither total dendritic complexity (Figure 1e) nor total dendritic length (Figure 1f) of APLP2-KO neurons was significantly affected (Student’s t-test, n.s.). Similarly to the results obtained for APLP2-KO neurons, analysis of APLP1-KO CA1 neurons revealed no significant differences in total dendritic length or dendritic branching (Additional file 1: Figure S1). These results indicate that neither lack of APLP2 nor of APLP1 causes major alterations in the neuronal architecture of mature CA1 pyramidal cells in organotypic hippocampal cultures.

### APP-KO neurons show reduced complexity of apical dendrites and an increased number of primary and secondary basal dendrites

**Figure 2:**
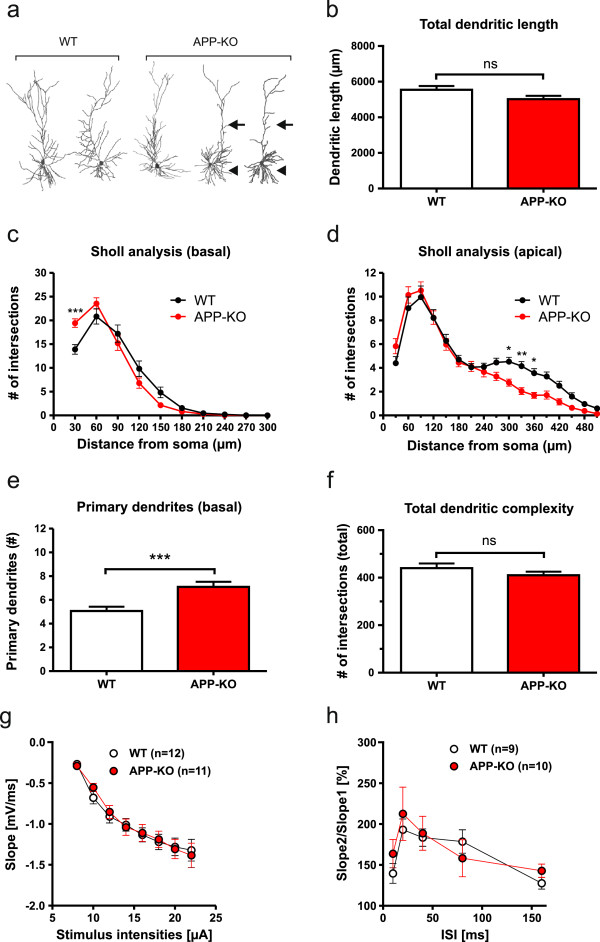
**Loss of APP affects morphology of hippocampal CA1 pyramidal neurons. (a)** Representative examples of 3D-reconstructed CA1 neurons from WT (left) and APP-KO mice (right). Note the differences in dendritic complexity: arrows indicate reduced complexity of mid-apical dendrites and arrowheads an increase in the number of primary basal dendrites in APP-KO neurons. **(b)** Total dendritic length in APP-KO neurons was slightly, but not significantly decreased when compared to WT neurons (Student’s t-test, p = 0.07). **(c, d)** Sholl analysis comparing basal **(c)** and apical **(d)** dendrites between WT and APP-KO neurons. **(c)** In basal dendrites of APP-KO neurons dendritic complexity was increased in regions proximal to the soma (30 μm distance; Repeated measure ANOVA with Bonferroni multiple comparison test, ***p ≤ 0.001). (**d**) Note that in apical dendrites of APP-KO cells dendritic complexity was highly reduced at 300 μm, 330 μm and 360 μm (Repeated measure ANOVA with Bonferroni multiple comparison test, *p ≤ 0.05, **p ≤ 0.01). **(e)** Comparison of the number of primary basal dendrites. Note that the number of primary basal dendrites is considerably increased in APP-KO (WT: 5.06 ± 0.37 versus APP-KO: 7.10 ± 0.43; Student’s t-test, ***p ≤ 0.001, WT data same as in Figure 1). **(f)** Total dendritic complexity was decreased, but did not reach significance likely due to the opposing effects on basal and apical dendrites. **(g)** Input-output-strength was analyzed in organotypic hippocampal cultures at DIV21-24, which revealed no alterations in basal synaptic transmission between WT and APP-KO mice. **(h)** Paired-pulse facilitation showed no significant differences between genotypes. **(g, h)** Student’s t-test, n = number of OHCs analyzed per genotype. **(b-f)** WT: n = 32 neurons/6 mice, APP-KO: n = 42 neurons/7 mice. All values represent mean ± SEM.

In contrast, APP-KO CA1 neurons displayed several distinct alterations of neuronal architecture, already apparent when inspecting reconstructed dendritic trees (Figure 2a). Although no overall significant differences in total dendritic length (see Figure 2b) and total dendritic complexity were detectable (see Figure 2f; Student’s t-test, n.s.), detailed Sholl analysis revealed a pronounced reduction of dendritic complexity in mid-apical regions of apical dendrites of APP-KO neurons as compared to WT CA1 neurons (Figure 2d; Repeated measure ANOVA, Genotype F_(1,72)_ = 4.293, p = 0.04, Bonferroni multiple comparison test: p < 0.05 for 300 μm, 330 μm, 360 μm). In addition, we observed a significantly increased number of primary (Figure 2e; Student’s t-test, p ≤ 0.001) and secondary basal dendrites (data not shown). Moreover, Sholl analysis revealed significantly increased branching in proximal regions (30 μm) of basal dendrites of APP-KO CA1 pyramidal cells (Figure 2c). Thus, in contrast to APLP-deficiency, lack of APP has major effects on the neuronal architecture of CA1 pyramidal cells.

Due to the perinatal lethality of APP/APLP2-DKO mice we used OHCs prepared at P0 in this study. To functionally characterize these preparations in more detail and to assess whether morphological defects might be related to compromised synaptic transmission, recordings of field potentials (fEPSPs) were performed (Figure 2g) in stratum radiatum corresponding to mid-apical regions of CA1 cells at a distance of ~100-200 μm from the soma. Field potentials could readily be measured, indicating functionally intact synaptic connections of the Schaffer collateral CA3/CA1 pathway. Compared to WT slices, APP-KO slices showed no significant differences in basal synaptic transmission (Figure 2g). In addition, we also studied short-term plasticity and analyzed paired-pulse facilitation (PPF) at various interstimulus intervals (see Figure 2h). PPF could readily be induced in both WT and APP-KO OHCs at all interstimulus intervals tested, indicating the functionality of presynaptic components in these preparations. No significant differences in PPF were detectable in APP-KO slices compared to WT slices (Figure 2h). Taken together these data suggest that branching defects observed in APP-KO mice are not secondary to major defects in basal synaptic transmission.

### APP/APLP2 double knockout mutants show additional branching defects as compared to single KO mutants

Although APLP2-KO neurons showed unaltered neuronal morphology we next assessed whether a redundant function of APLP2 for neuronal architecture might have been masked and compensated for by the presence of APP. Thus, to circumvent functional compensation, we studied neuronal morphology in APP/APLP2-DKO cultures in comparison to APP-KO and APLP2-KO control cultures and asked whether the combined loss of APP and APLP2 would lead to additional defects beyond those already seen in APP-KO mice.

**Figure 3:**
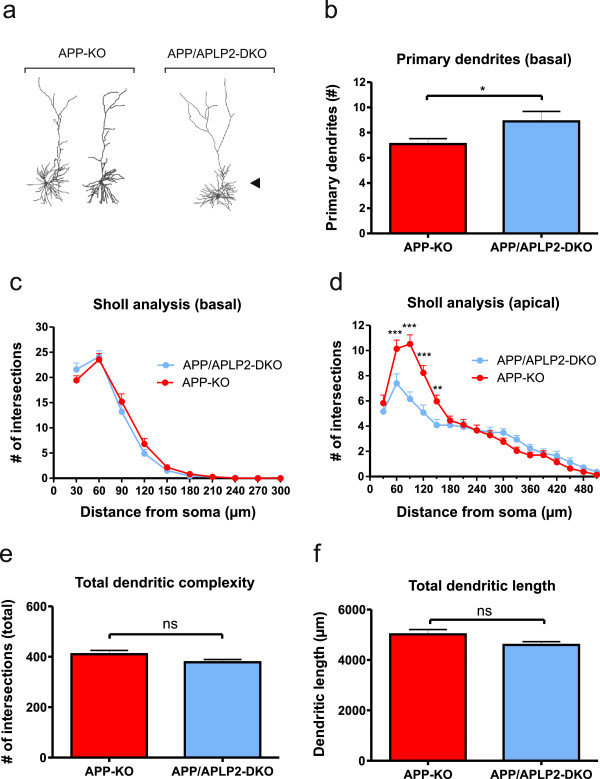
**Pyramidal cells from combined APP/APLP2-DKO neurons show additional defects in dendritic complexity compared to APP-KO single mutants.(a)** Representative example of 3D-reconstructed CA1 neurons from APP-KO mice (left) and APP/APLP2-DKO mice (right). Note the differences in dendritic complexity: arrowhead indicates an increase in the number of primary basal dendrites in APP/APLP2-DKO neurons. **(b)** Comparison of the number of primary basal dendrites revealed an increase in APP/APLP2-DKO neurons when compared to neurons from APP-KO mice (APP-KO: 7.10 ± 0.43 versus APP/APLP2-DKO 8.89 ± 0.79; Student’s t-test, *p ≤ 0.05, APP-KO data same as in Figure 2). **(c)** The branching pattern of basal dendrites from APP/APLP2-DKO mice resembles that of APP-KO neurons (Repeated measure ANOVA with Bonferroni multiple comparison test, n.s.) whereas **(d)** apical dendrites of APP/APLP2-DKO neurons are characterized by an additional branching defect close to the soma (60 μm, 90 μm, 120 μm, 150 μm; Repeated measure ANOVA with Bonferroni multiple comparison test, **p ≤ 0.01, ***p ≤ 0.001). **(e, f)** Total dendritic complexity **(e)** and total dendritic length **(f)** was not affected in APP/APLP2-DKO neurons as compared to neurons from APP-KO mice (Student’s t-test, n.s.; APP-KO: n = 42 neurons/7 mice, APP/APLP2-DKO: n = 37 neurons/6 mice. All values represent mean ± SEM.

In APP/APLP2-DKO neurons we found a further significant increase in the number of primary basal dendrites compared to APP-KO neurons (Figure 3b; Student’s t-test, p ≤ 0.05). Overall, Sholl analysis of basal dendrites revealed a branching pattern highly similar to that of APP-KO single mutants (Figure 3c, Repeated measure ANOVA with Bonferroni multiple comparison test, Genotype F_(1,77)_ = 0.1594, p = 0.69, n.s.). Interestingly, however, Sholl analysis of apical dendrites from DKO neurons lacking both APP and APLP2 showed prominent additional branching defects in proximal apical dendrites as compared to APP-deficient single KO mice (Figure 3d; Repeated measure ANOVA, Genotype F_(1,77)_ = 5.149; p = 0.03, Bonferroni multiple comparison test: p ≤ 0.05 for 60 μm, 90 μm, 120 μm, 150 μm). Likewise, APP/APLP2-DKO also showed a similar increase in branching of proximal apical dendrites when compared to APLP2-KO single mutant (see Additional file 1: Figure S2d; Repeated measure ANOVA, Genotype F_(1,60)_ = 9.155, p = 0.004, Bonferroni multiple comparison test: p ≤ 0.001 for 90).

The additional branching defects of APP/APLP2-DKO neurons as compared to APLP2-KO neurons were also reflected in a now significantly reduced total dendritic complexity and total dendritic length (Additional file 1: Figure S2e, f; Student’s t-test, p ≤ 0.05). Taken together these data indicate functional redundancy and suggests that both APP and APLP2 are required for normal neuronal morphology.

### APP-KO neurons show a highly reduced spine density that is not further decreased by an additional lack of APLP2

**Figure 4:**
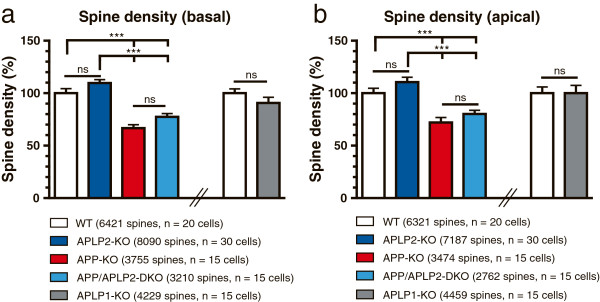
**Comparative analysis of APP/APLP mutant mice reveals a reduction in spine density only in APP and APP/APLP2-DKO neurons. (a, b)** Spine density counts were performed for either basal **(a)** or mid-apical **(b)** dendrites of CA1 neurons of the indicated genotypes and values expressed relative to APP-WT dendrites set as 100%. **(a, b)** Basal and apical dendrites from APP-KO and APP/APLP-DKO both show significant reductions in spine density, as compared to either WT or APLP2-KO mice. In contrast, APLP2-KO and APLP1-KO were unaffected. Note that spine density of APP/APLP2-DKO dendrites was not significantly different from that of APP-KO dendrites. Next, we compared WT and APLP1-KO neurons. Spine density of APLP1-KO neurons was comparable to WT neurons. Spine density was determined using n = 15-30 neurons/ genotype from 5-12 mice per genotype. All Values represent mean ± SEM, One-way ANOVA with Bonferroni multiple comparison test. Asterisks indicate statistically significant differences; ***p ≤ 0.001.

Next, we performed spine density counts on reconstructed basal dendrites and in mid-distal portions of apical dendrites (roughly corresponding to the region that showed reduced branching in APP-KO and APP/APLP2-DKO neurons) of all single and combined knockout mutants (Figure 4). Remarkably, we found a highly reduced (by 31.1 ± 2.2%) spine density in APP-KO neurons both in apical dendritic regions and for basal dendrites of APP-KO neurons as compared to WT neurons (see Figure 4a,b; One-way ANOVA, with Bonferroni multiple comparison test, p ≤ 0.001). Although neurons biolistically transfected with fEGFP showed no signs of degeneration such as swelling or retraction bulbs we wanted to further substantiate our findings using an independent method and mouse line for analysis. We generated entorhino-hippocampal slice cultures of APP-KO mice crossed with a transgenic Thy1-GFP mouse line (M line) [33] and imaged side branches of mid-apical dendrites of GFP-labeled CA1 neurons. In these preparations a similar reduction of dendritic spine density was observed (by 24.2 ± 3.5%, Student’s t-test, p ≤ 0.01).

In contrast to APP-KO pyramidal cells, we were unable to detect significant differences in spine density in basal or apical dendrites of APLP2-KO mice (Figure 4a, b; One-way ANOVA with Bonferroni multiple comparison test, p > 0.05). To determine whether APLP2 has a similar and potentially redundant role for spine density as observed for dendritic complexity and arborization, we also determined spine density in combined APP/APLP2-DKO neurons. In double mutants, we detected again a prominent reduction in spine density compared to dendrites of APLP2-KO and WT neurons (Figure 4a,b; One-way ANOVA with Bonferroni post hoc test, p ≤ 0.001). Spine density was, however, not further reduced by an additional lack of APLP2 in APP/APLP2-DKO neurons, as evidenced by a similar, not significantly different reduction in spine density in DKO neurons as compared to APP-KO neurons (Figure 4a,b). Next, we also determined spine density in APLP1-KO versus WT CA1 neurons. As seen for APLP2-KO neurons, spine density was not significantly affected by the lack of APLP1 (Figure 4a, b).

### Expression of APPsα prevents spine density deficits and altered spine type distribution

**Figure 5:**
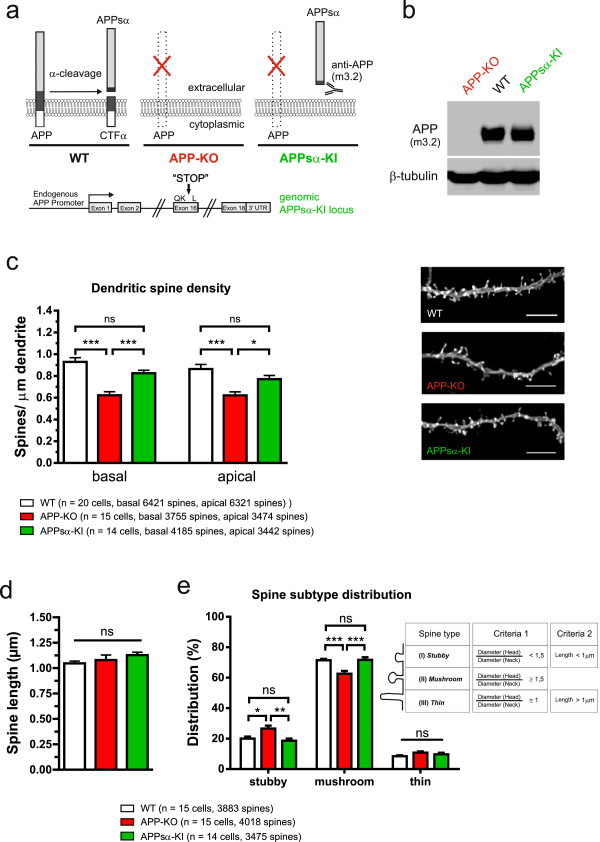
**APP-KO neurons exhibit alterations in spine density and spine type distribution that are rescued in CA1 neurons of APPsα-KI mice. (a)** Scheme depicting the genotypes analyzed and selected proteolytic APP fragments. Note that APPsα-KI neurons lack transmembrane APP and express solely the secreted APPsα ectodomain, recognized by m3.2 antibody. Bottom: scheme of the modified genomic APPsα-KI locus. **(b)** Western blot analysis of soluble APPs expressed in APPsα-KI brain probed with the APPsα-specific antibody m3.2. APP-KO brain was used as a negativ control, β-tubulin staining as a loading control **(c)** left panel: Spine density of basal and apical dendrites of APP-KO CA1 neurons is severely reduced compared to WT neurons (One-way ANOVA with Bonferroni multiple comparison, ***p ≤ 0.001) whereas no significant alterations were detectable for APPsα-KI neurons (One-way ANOVA with Bonferroni multiple comparison, n.s.). Number of neurons used for spine density determination: WT n = 20, APP-KO n = 15, APPsα-KI n = 14. Right panel: Representative images of apical dendrites from the indicated genotypes (Scale bar: 5 μm). **(d)** Spine length was not significantly altered between WT, APP-KO and APPsα-KI (One-way ANOVA, n.s.). **(e)** Comparison of the relative frequency of spine types. Note that APP-KO neurons show a significant reduction in the number of mushroom spines and increased numbers of stubby spines (see results for details). Spine type distribution of APPsα-KI neurons was not significantly different from WT neurons. For spine type distribution analysis the number of neurons per genotype was: WT n = 15, APP-KO n = 15, APPsα-KI n = 14. Values represent mean ± SEM; One-way ANOVA with Bonferroni multiple comparison test, *p < 0.05, **p < 0.01, ***p < 0.001.

APP family proteins are known to form cis- and trans-dimers on the cell surface [36] and trans-cellular adhesion of APP family proteins may induce hemisynapses *in vitro*, as APP overexpression in HEK cells was shown to induce presynaptic specialization in co-cultured hippocampal neurons [37]. In line with a synaptotrophic role of APP it has been demonstrated that overexpression of recombinant human APP (huAPP) in dissociated hippocampal neurons increases spine density [17]. Our finding that APP-KO neurons exhibit reduced spine density indicated that a lack of transmembrane APP or the lack of one of the proteolytic APP fragments causes this effect. To gain further mechanistic insight we therefore investigated spine density in OHCs prepared from our previously generated APPsα knockin (KI) mice [15]. In these mice we had used gene targeting in ES cells to introduce a stop codon into exon 16 of the endogenous mouse APP locus just behind the α-secretase cleavage site. Thus, APPsα-KI mice lack full length APP and express instead solely the secreted APPsα ectodomain (see Western blot Figure 5b) albeit at a somewhat reduced level (60% of WT level, as assessed by qPCR; see [15]) under control of the endogenous APP promoter (Figure 5a), throughout development including the time of neuronal differentiation and synaptogenesis. Strikingly, spine density of APPsα-KI CA1 neurons was not significantly different from that of WT neurons on either basal dendrites or mid-apical segments of apical dendrites, indicating that the APP C-terminus is dispensable and that transmembrane APP isoforms are not essential to mediate normal spine counts (see Figure 5c, One-way ANOVA, basal: Genotype F_(2,47)_ = 20.25, p < 0.001, apical: Genotype F_(2,47)_ = 10.46, p = 0.0002; Bonferroni multiple comparison for basal and apical: WT or APPsα-KI vs. APP-KO p ≤ 0.001, WT vs. APPsα-KI n.s.).

We also compared the size and form of dendritic spines, as changes in these parameters are expected to have a major impact on biochemical signaling and electrophysiological properties of synapses [38,39]. Three major spine types can be distinguished by morphological criteria: stubby, thin and mushroom spines. This classification is based on measurements of spine length and additionally the ratio between the spine head and spine neck diameter providing objective criteria (see Figure 5e and materials and methods) to classify the great variety of spine morphologies [29-31]. Spine type analysis (see Figure 5e) revealed that APP-KO CA1 neurons show significantly fewer mushroom-type spines (62.8 ± 0.4% versus 71.5 ± 0.4%; n = 14-15 neurons/genotype and more than 4300 spines/genotype, One-way ANOVA with Bonferroni multiple comparison test, p ≤ 0.001) and a corresponding increase in stubby spines (26.6 ± 0.4% versus 20.1 ± 0.3%, One-way ANOVA with Bonferroni multiple comparison test, p ≤ 0.05).

Again, for APPsα-KI neurons, however, no significant alterations in spine type distribution were detectable as compared to WT neurons, suggesting that APPsα is sufficient for signals mediating normal spine type distribution (Figure 5e; stubby spines: ANOVA F_(2,41)_ = 7.266, p = 0.002; mushroom spines: ANOVA F_(2,41)_ = 11.43, p = 0.0001; thin spines: ANOVA F_(2,41)_ = 2.064, p = 0.1399). No significant alterations in spine length were detectable in any of the genotypes studied (Figure 5d; One-way ANOVA, F_(2,41)_ = 1.254, p = 0.30, n.s.; WT (1.047 ± 0.03 μm), APP-KO (1.079 ± 0.05 μm) and APPsα-KI (1.128 ± 0.03 μm)).

The role of APPsα for dendritic branching appears to be more complex. Although APPsα-KI mice showed a rescue of dendritic branching of distal apical segments (Additional file 1: Figure S3), i.e. the region where we had observed a reduced dendritic complexity of APP-KO neurons, no rescue was observed for basal dendrites (Additional file 1: Figure S3). This selective effect of APPsα on distal apical dendrites contrasts with the more general role of APPsα for spine density on both basal and apical dendrites of CA1 pyramidal cells. Collectively, these studies of CA1 pyramidal cells in OHCs revealed a combined role of APP and APLP2 for dendritic architecture already at early developmental stages and a unique function of secreted APPs, in particular APPsa, for spine density.

## Discussion

The analysis of APP physiological functions has been complicated by functional redundancy between APP and the structurally related and similarly processed APLPs. We therefore conducted a systematic comparative analysis of neuronal morphology in single and combined APP/APLP knockout mutants. One of the key findings of this study is that both APP and APLP2 are required for normal dendritic complexity of CA1 pyramidal cells. In addition, we show that APP serves a unique, non-redundant physiological function for spine structure, as evidenced by a striking decrease in spine density selectively in APP-KO mice, in particular a reduction in the proportion of mushroom spines thought to represent mature synapses. Mechanistically, we demonstrate using a genetic approach that the endogenously produced secreted APPsα fragment is sufficient to mediate signals that result in normal spine densities in the absence of transmembrane APP isoforms.

APP family proteins are highly expressed in neurons with peak levels paralleling synaptogenesis [40,41] and are enriched in motile tips of growth cones where APP colocalizes with the adaptor protein Fe65 in actin-rich lamellipodia [40,42]. In addition, APP and APLPs have been shown to interact with extracellular matrix components such as heparin, HPSGs, laminin and collagen via their conserved N-terminal domains [43-45]. Consistent with a role in cell-substrate and/or cell-cell adhesion we had previously shown that triple knockout of all three APP/APLP proteins [46] or *in utero* knockdown of APP/APLPs lead to neuronal migration defects during cortical development [45,46].

Several reports in neuroblastoma cells or dissociated neurons have previously linked APP expression to neurite outgrowth [45,47-55]. Hippocampal neurons deficient for APP showed initially reduced neurite outgrowth, whereas after prolonged culture, elongation of the longest neurite was reported [45,47]. However, *in vitro* studies using dissociated neurons may be highly variable, due to effects of different cell densities and/or concentrations of secreted factors [17,19]. Thus, we intended to re-examine the role of APP for neuronal morphology and in addition to study systematically the specific or potentially redundant role of APLP1 and APLP2 in organotypic hippocampal cultures (OHCs), a well established preparation closely resembling the *in vivo* situation with regard to neuronal connectivity and cell types (e.g. excitatory and inhibitory neurons, glia) that allows to study mature neurons [22,56-58]. Importantly, using OHCs we could also circumvent the perinatal lethality of APP/APLP2 DKO mutants.

### Combined role of APP and APLP2 for dendritic complexity

Amongst the APP family members APP has a prominent role for dendritic architecture as the knockout of APP alone strongly affected morphology of mature CA1 neurons, while lack of either APLP1 or APLP2 did not lead to alterations. However, functional redundancy can only be assessed in combined mutants, as previously demonstrated for the lethality and defects in neuromuscular or cortical development observed in double and triple knockouts [20,21,46]. Indeed, in APP/APLP2-DKO neurons we found striking additional defects in the dendritic complexity of apical dendrites as compared to single APP-KO mutants. These findings reveal a novel function of APLP2 for neuronal morphology in the hippocampus. Further studies are needed to investigate a potentially similar role for APLP1, and can only be fully addressed upon generation of conditional APP/APLP triple mutants (see e.g. [59]). *In vitro* studies have implicated multiple domains of APP in neurite outgrowth including APP transmembrane isoforms, the secreted APP ectodomain, or CTFs and several mechanisms have been proposed [45,49,54,55,60,61]. In light of our findings that both APP and APLP2 are important for neuronal morphology in OHCs it appears likely that these functions are mediated by conserved APP/APLP protein domains and may involve APP/APLP mediated adhesion or signaling [36,55,62]. This view is consistent with our previous studies of the neuromuscular junction, indicating that both secreted APPsα and transmembrane isoforms of APP and APLP2 are required for normal neuromuscular junction development and function [35]. As APP has also been implicated in axonal outgrowth [45,51,52,55] it is conceivable that alterations in dendritic complexity of APP-KO and APP/APLP2-DKO neurons might also be related to changes in axonal projections leading to reduced synaptic input e.g. in distal regions of CA1 apical dendrites. Our data also suggest that morphological postsynaptic defects occur despite the lack of major presynaptic defects and are thus not secondary to major defects in transmitter release. In fact, similar fEPSP values in mid-apical regions between WT and APP-KO correspond well to unaltered dendritic branching in these regions. In APP-KO mice dendritic branching defects and reduced spine density of apical dendrites was restricted to further distal dendritic segments (300, 330 and 360 μm), at a distance from the soma at which extracellular EPSP values were too small to be detectable. So far, our data on EPSP responses, short-term plasticity (this study) and LTP (see [35] and data not shown) have not revealed significant differences between WT and APP-KO OHCs. This might also be due to the increased experimental variability of the OHC preparations that reduces sensitivity of the functional readout and precludes the detection of more subtle defects. Future studies focusing on the relationship between the morphological defects and neuronal function will have to shed light on this issue.

### Endogenously produced APPsα is sufficient for normal spine density and spine type distribution

The second key finding was the surprisingly unique role of APP for dendritic spine density, as evidenced by a substantial reduction in spine counts in APP-KO neurons and no detectable alterations in either APLP1-KO or APLP2-KO dendrites. Importantly, even a combined lack of APP and APLP2 (in APP/APLP2-DKO cultures) did not reveal a role of APLP2 for spine density. These data are further supported by our previous electrophysiological studies in hippocampal slices from aged mice, which indicated that lack of APP results in LTP deficits [15], whereas lack of APLP2 does neither affect basal synaptic transmission nor synaptic short- or long-term plasticity, even in aged mice [35]. In this regard, it is important to note that previous *in vitro* studies using dissociated APP-KO neurons led to conflicting results with an about twofold increase in functional synapses in low density autaptic cultures of APP-KO neurons [19] contrasting with decreased spine densities in bulk cultures from APP-KO mice [63] or upon shRNA knockdown of APP [17]. The reason for these conflicting data is presently unclear but might be related to differences in cell density and the fact that dissociated cultures only imperfectly model physiological concentrations of soluble factors and/or neuronal connectivity.

Our finding that lack of APP leads to highly reduced spine counts in OHCs indicates that APP or any of the various proteolytic fragments such as Aβ, secreted APPs, CTFs or AICD is required for normal spine numbers. Indeed, several hypothesis how APP may affect spine density had been proposed, based on *in vitro* systems or pharmacological intervention *in vivo*. Using dissociated neurons Lee and coworkers suggested that the reduced spine density resulting from APP knockdown *in vitro* may be due to a lacking interaction of cell surface APP with the extracellular matrix protein Reelin [17]. In contrast, another study observed increased spine densities (as assessed by two photon *in vivo* imaging) in apical tufts of cortical neurons of adult (4-6 month old) APP-KO mice [18,19]. Given that γ-secretase inhibitors reduced spine density of WT neurons, but had no effect on APP-KO neurons, it was proposed that the inability to produce Aβ or AICD may increase synapse formation in APP-KO neurons [18,19]. However, interpretation of pharmacological data is difficult due to effects that may be mediated by other targets, which is particularly critical for γ−secretase with a multitude of additional substrates besides APP [2]. In this regard, the same γ-secretase inhibitor (DAPT) when applied to dissociated WT neurons, had previously been found to increase synapse numbers (as opposed to the decrease found in adult cortex), whereas APP-KO neurons were again not affected by DAPT treatment [19].

In this study, we therefore choose a genetic approach to directly address the physiological role of APP family members in intact hippocampal tissue and demonstrate that the endogenously produced secreted APPsα ectodomain is sufficient for normal spine density in mature CA1 neurons even in the absence of transmembrane APP. Our data obtained with hippocampal tissue are further supported by a recent *in vitro* study showing that conditioned media containing APPsα can to some extent (by about 50%) restore spine density deficits of cultured APP-KO neurons [63]. In turn, our findings also imply that autocrine or paracrine APPsα signaling, important for spine formation and/or maintenance, involves a so far unknown receptor distinct from APP itself. Collectively, we report a unique non redundant role of APP and APPsα for spine density of CA1 neurons and a synergistic role of APP and APLP2 for dendritic morphology.

### Role of APP and APPsα in the adult brain, for aging and AD pathogenesis

Additional evidence indicates a requirement of APP for neuronal architecture and function also in adult mice. A recent study described reduced dendritic complexity and a moderate reduction in spine density in CA1 neurons of aged (12-15 month old) APP-KO animals [63], that was associated with pronounced deficits in LTP of aged APP-KO mice [14,15,64], which we had previously shown to be rescued in APPsα-KI mice [15]. Together, this indicates a dual role of APP for spine structure: an early requirement of APP at stages of spine formation/maturation and also for the maintenance of spines during aging. Further support for a synaptotrophic role of APP and APPsα comes from transgenic mice with moderate overexpression of human WT APP [65], or indirect up-regulation of APPsα by transgenic expression of the α-secretase ADAM10 [66], that is enriched at synaptic contacts [67], which all lead to increased synaptic density. In Tg2576 mice expression of mutant huAPP increased spine density in CA1 and cortical neurons of young mice prior to plaque deposition possibly via APPsα, whereas spine density was decreased in aged animals, presumably due to Aβ-mediated synaptotoxic effects [16,17]. In addition, oligomeric forms of Aβ have been reported to compromise synaptic function in Tg2576 mice even before plaque development [68]. Thus, different APP fragments (e.g. APPsα and Aβ) likely mediate opposing functions for synapse formation or maintenance that are highly relevant not only for normal brain physiology but also AD pathogenesis. As there is a shift towards the amyloidogenic pathway in AD reduced levels of APPsα may contribute to AD pathogenesis [5]. Indeed, decreased levels of APPsα levels and/or ADAM10 activity (the major the α-secretase) have been reported in the cerebrospinal fluid (CSF) of AD patients [69-74]. Moreover, lowered levels of CSF APPsα are correlated with poor memory performance in aged WT rats [75]. Intriguingly, a recent study indicated that APPsα can also directly modulate APP processing by reducing BACE activity, and thereby lower Aβ levels in cells and in AD model mice [76]. Irrespective of whether there is a lack-of-function component in AD pathogenesis caused by diminished APPsα production, the well-established functions of APPsα in neuroprotection (see e. g. [77] and review by [1]), synaptic plasticity [15,35,78,79] and our data from the present study suggest that enhancing/restoring of APPsα levels may be beneficial to counteract and alleviate early AD related symptoms including deficits in spine density.

### Notes

We are grateful to Diane Mundil for preparing organotypic cultures for electrophysiological analysis. We thank Dr. Gaby Schneider, Goethe University, for helpful suggestions concerning the statistics. The monoclonal anti-APP antibody m3.2 was kindly provided by Dr. Paul Mathews (NY, USA). We also like to thank the Nikon Imaging Center (University of Heidelberg) and Ulrike Engel, Christian Ackermann, Nicolas Dross and Pete Bankhead for support with confocal microscopy and image analysis.

## Competing interests

The authors declare no competing financial interests.

## Supplementary Material

Additional file 1: Figure 1, Figure2, Figure 3 Comparative analysis of single and combined APP/APLP knockouts reveals reduced spine density in APP-KO mice that is prevented by APPsα expression. Click here for file

## References

[B1] Kogel D Deller T Behl C Roles of amyloid precursor protein family members in neuroprotection, stress signaling and aging Exp Brain Res 2012 2 471 479 10.1007/s00221-011-2932-4 10.1007/s00221-011-2932-4 22086493

[B2] Lichtenthaler SF Haass C Steiner H Regulated intramembrane proteolysis–lessons from amyloid precursor protein processing J Neurochem 2011 2 779 796 10.1111/j.1471-4159.2011.07248.x 10.1111/j.1471-4159.2011.07248.x 21413990

[B3] Prox J Rittger A Saftig P Physiological functions of the amyloid precursor protein secretases ADAM10, BACE1, and Presenilin Exp Brain Res 2012 2 331 341 10.1007/s00221-011-2952-0 10.1007/s00221-011-2952-0 22120156

[B4] Endres K Fahrenholz F Upregulation of the alpha-secretase ADAM10--risk or reason for hope? FEBS J 2010 2 1585 1596 EJB7566 10.1111/j.1742-4658.2010.07566.x 10.1111/j.1742-4658.2010.07566.x 20136654

[B5] Endres K Fahrenholz F Regulation of alpha-secretase ADAM10 expression and activity Exp Brain Res 2012 2 343 352 10.1007/s00221-011-2885-7 10.1007/s00221-011-2885-7 21969210

[B6] Aydin D Weyer SW Müller UC Functions of the APP gene family in the nervous system: insights from mouse models Exp Brain Res 2012 2 423 434 10.1007/s00221-011-2861-2 10.1007/s00221-011-2861-2 21931985

[B7] Jacobsen KT Iverfeldt K Amyloid precursor protein and its homologues: a family of proteolysis-dependent receptors Cell Mol Life Sci 2009 2 2299 2318 10.1007/s00018-009-0020-8 10.1007/s00018-009-0020-8 19333550 PMC11115575

[B8] Müller UC Zheng H Physiological functions of APP family proteins Cold Spring Harb Perspect Med 2012 2 a006288 10.1101/cshperspect.a006288 a006288 22355794 10.1101/cshperspect.a006288 PMC3281588

[B9] Müller U Cristina N Li ZW Wolfer DP Lipp HP Rülicke T Brandner S Aguzzi A Weissmann C Behavioral and anatomical deficits in mice homozygous for a modified beta-amyloid precursor protein gene Cell 1994 2 755 765 10.1016/0092-8674(94)90066-3 8001115

[B10] Zheng H Jiang M Trumbauer ME Sirinathsinghji DJ Hopkins R Smith DW Heavens RP Dawson GR Boyce S Conner MW Stevens KA Slunt HH Sisoda SS Chen HY Van der Ploeg LH beta-Amyloid precursor protein-deficient mice show reactive gliosis and decreased locomotor activity Cell 1995 2 525 531 10.1016/0092-8674(95)90073-X 7758106

[B11] Li ZW Stark G Gotz J Rülicke T Gschwind M Huber G Müller U Weissmann C Generation of mice with a 200-kb amyloid precursor protein gene deletion by Cre recombinase-mediated site-specific recombination in embryonic stem cells Proc Natl Acad Sci U S A 1996 2 6158 6162 10.1073/pnas.93.12.6158 8650236 PMC39206

[B12] Dawson GR Seabrook GR Zheng H Smith DW Graham S O’Dowd G Bowery BJ Boyce S Trumbauer ME Chen HY Van der Ploeg LH Sirinathsinghji DJ Age-related cognitive deficits, impaired long-term potentiation and reduction in synaptic marker density in mice lacking the beta-amyloid precursor protein Neuroscience 1999 2 1 13 S0306-4522(98)00410-2 10.1016/S0306-4522(98)00410-2 10188929

[B13] Magara F Müller U Li ZW Lipp HP Weissmann C Stagljar M Wolfer DP Genetic background changes the pattern of forebrain commissure defects in transgenic mice underexpressing the beta-amyloid-precursor protein Proc Natl Acad Sci U S A 1999 2 4656 4661 10.1073/pnas.96.8.4656 10200318 PMC16388

[B14] Seabrook GR Smith DW Bowery BJ Easter A Reynolds T Fitzjohn SM Morton RA Zheng H Dawson GR Sirinathsinghji DJ Davies CH Collingridge GL Hill RG Mechanisms contributing to the deficits in hippocampal synaptic plasticity in mice lacking amyloid precursor protein Neuropharmacology 1999 2 349 359 S0028390898002044 10.1016/S0028-3908(98)00204-4 10219973

[B15] Ring S Weyer SW Kilian SB Waldron E Pietrzik CU Filippov MA Herms J Buchholz C Eckman CB Korte M Wolfer DP Müller UC The secreted beta-amyloid precursor protein ectodomain APPs alpha is sufficient to rescue the anatomical, behavioral, and electrophysiological abnormalities of APP-deficient mice J Neurosci 2007 2 7817 7826 27/29/7817 10.1523/JNEUROSCI.1026-07.2007 10.1523/JNEUROSCI.1026-07.2007 17634375 PMC6672885

[B16] Jung CK Herms J Role of APP for dendritic spine formation and stability Exp Brain Res 2012 2 463 470 10.1007/s00221-011-2939-x 10.1007/s00221-011-2939-x 22094714

[B17] Lee KJ Moussa CE Lee Y Sung Y Howell BW Turner RS Pak DT Hoe HS Beta amyloid-independent role of amyloid precursor protein in generation and maintenance of dendritic spines Neuroscience 2010 2 344 356 S0306-4522(10)00645-7 10.1016/j.neuroscience.2010.04.078 10.1016/j.neuroscience.2010.04.078 20451588 PMC2900520

[B18] Bittner T Fuhrmann M Burgold S Jung CK Volbracht C Steiner H Mitteregger G Kretzschmar HA Haass C Herms J Gamma-secretase inhibition reduces spine density in vivo via an amyloid precursor protein-dependent pathway J Neurosci 2009 2 10405 10409 29/33/10405 10.1523/JNEUROSCI.2288-09.2009 10.1523/JNEUROSCI.2288-09.2009 19692615 PMC6665795

[B19] Priller C Mitteregger G Paluch S Vassallo N Staufenbiel M Kretzschmar HA Jucker M Herms J Excitatory synaptic transmission is depressed in cultured hippocampal neurons of APP/PS1 mice Neurobiol Aging 2009 2 1227 1237 S0197-4580(07)00416-2 10.1016/j.neurobiolaging.2007.10.016 10.1016/j.neurobiolaging.2007.10.016 18077058

[B20] Heber S Herms J Gajic V Hainfellner J Aguzzi A Rülicke T von Kretzschmar H von Koch C Sisodia S Tremml P Lipp HP Wolfer DP Müller U Mice with combined gene knock-outs reveal essential and partially redundant functions of amyloid precursor protein family members J Neurosci 2000 2 7951 7963 11050115 10.1523/JNEUROSCI.20-21-07951.2000 PMC6772747

[B21] Wang P Yang G Mosier DR Chang P Zaidi T Gong YD Zhao NM Dominguez B Lee KF Gan WB Zheng H Defective neuromuscular synapses in mice lacking amyloid precursor protein (APP) and APP-Like protein 2 J Neurosci 2005 2 1219 1225 10.1523/JNEUROSCI.4660-04.2005 15689559 PMC6725967

[B22] Stoppini L Buchs PA Müller D A simple method for organotypic cultures of nervous tissue J Neurosci Methods 1991 2 173 182 0165-0270(91)90128-M 10.1016/0165-0270(91)90128-M 1715499

[B23] Vlachos A Bas Orth C Schneider G Deller T Time-lapse imaging of granule cells in mouse entorhino-hippocampal slice cultures reveals changes in spine stability after entorhinal denervation J Comp Neurol 2012 2 1891 1902 10.1002/cne.23017 10.1002/cne.23017 22134835

[B24] O’Brien JA Holt M Whiteside G Lummis SC Hastings MH Modifications to the hand-held Gene Gun: improvements for in vitro biolistic transfection of organotypic neuronal tissue J Neurosci Methods 2001 2 57 64 S0165-0270(01)00457-5 10.1016/S0165-0270(01)00457-5 11640958

[B25] O’Brien JA Lummis SC Biolistic transfection of neuronal cultures using a hand-held gene gun Nat Protoc 2006 2 977 981 nprot.2006.145 10.1038/nprot.2006.145 10.1038/nprot.2006.145 17406333 PMC2649370

[B26] Sholl DA Dendritic organization in the neurons of the visual and motor cortices of the cat J Anat 1953 2 387 406 13117757 PMC1244622

[B27] Holtmaat A Bonhoeffer T Chow DK Chuckowree J De Paola V Hofer SB Hübener M Keck T Knott G Lee WC Mostany R Mrsic-Flogel TD Nedivi E Portera-Cailliau C Svoboda K Trachtenberg JT Wilbrecht L Long-term, high-resolution imaging in the mouse neocortex through a chronic cranial window Nat Protoc 2009 2 1128 1144 nprot.2009.89 10.1038/nprot.2009.89 10.1038/nprot.2009.89 19617885 PMC3072839

[B28] Peters A Kaiserman-Abramof IR The small pyramidal neuron of the rat cerebral cortex. The perikaryon, dendrites and spines Am J Anat 1970 2 321 355 10.1002/aja.1001270402 10.1002/aja.1001270402 4985058

[B29] Harris KM Jensen FE Tsao B Three-dimensional structure of dendritic spines and synapses in rat hippocampus (CA1) at postnatal day 15 and adult ages: implications for the maturation of synaptic physiology and long-term potentiation J Neurosci 1992 2 2685 2705 1613552 10.1523/JNEUROSCI.12-07-02685.1992 PMC6575840

[B30] Koh IY Lindquist WB Zito K Nimchinsky EA Svoboda K An image analysis algorithm for dendritic spines Neural Comput 2002 2 1283 1310 10.1162/089976602753712945 10.1162/089976602753712945 12020447

[B31] Zagrebelsky M Holz A Dechant G Barde YA Bonhoeffer T Korte M The p75 neurotrophin receptor negatively modulates dendrite complexity and spine density in hippocampal neurons J Neurosci 2005 2 9989 9999 25/43/9989 10.1523/JNEUROSCI.2492-05.2005 10.1523/JNEUROSCI.2492-05.2005 16251447 PMC6725571

[B32] von Koch CS Zheng H Chen H Trumbauer M Thinakaran G van der Ploeg LH Price DL Sisodia SS Generation of APLP2 KO mice and early postnatal lethality in APLP2/APP double KO mice Neurobiol Aging 1997 2 661 669 10.1016/S0197-4580(97)00151-6 9461064

[B33] Feng G Mellor RH Bernstein M Keller-Peck C Nguyen QT Wallace M Nerbonne JM Lichtman JW Sanes JR Imaging neuronal subsets in transgenic mice expressing multiple spectral variants of GFP Neuron 2000 2 41 51 S0896-6273(00)00084-2 10.1016/S0896-6273(00)00084-2 11086982

[B34] Morales-Corraliza J Mazzella MJ Berger JD Diaz NS Choi JH Levy E Matsuoka Y Planel E Mathews PM In vivo turnover of tau and APP metabolites in the brains of wild-type and Tg2576 mice: greater stability of sAPP in the beta-amyloid depositing mice PLoS One 2009 2 e7134 10.1371/journal.pone.0007134 10.1371/journal.pone.0007134 19771166 PMC2741602

[B35] Weyer SW Klevanski M Delekate A Voikar V Aydin D Hick M Filippov M Drost N Schaller KL Saar M Vogt MA Gass P Samanta A Jäschke A Korte M Wolfer DP Caldwell JH Müller UC APP and APLP2 are essential at PNS and CNS synapses for transmission, spatial learning and LTP EMBO J 2011 2 2266 2280 emboj2011119 10.1038/emboj.2011.119 10.1038/emboj.2011.119 21522131 PMC3117640

[B36] Soba P Eggert S Wagner K Zentgraf H Siehl K Kreger S Löwer A Langer A Merdes G Paro R Masters CL Müller U Kins S Beyreuther K Homo- and heterodimerization of APP family members promotes intercellular adhesion Embo J 2005 2 3624 3634 10.1038/sj.emboj.7600824 16193067 PMC1276707

[B37] Wang Z Wang B Yang L Guo Q Aithmitti N Songyang Z Zheng H Presynaptic and postsynaptic interaction of the amyloid precursor protein promotes peripheral and central synaptogenesis J Neurosci 2009 2 10788 10801 29/35/10788 10.1523/JNEUROSCI.2132-09.2009 10.1523/JNEUROSCI.2132-09.2009 19726636 PMC2757256

[B38] Yuste R Bonhoeffer T Morphological changes in dendritic spines associated with long-term synaptic plasticity Annu Rev Neurosci 2001 2 1071 1089 10.1146/annurev.neuro.24.1.107124/1/1071 10.1146/annurev.neuro.24.1.1071 11520928

[B39] Konur S Rabinowitz D Fenstermaker VL Yuste R Systematic regulation of spine sizes and densities in pyramidal neurons J Neurobiol 2003 2 95 112 10.1002/neu.10229 10.1002/neu.10229 12838576

[B40] Szodorai A Kuan YH Hunzelmann S Engel U Sakane A Sasaki T Takai Y Kirsch J Müller U Beyreuther K Brady S Morfini G Kins S APP anterograde transport requires Rab3A GTPase activity for assembly of the transport vesicle J Neurosci 2009 2 14534 14544 29/46/14534 10.1523/JNEUROSCI.1546-09.2009 10.1523/JNEUROSCI.1546-09.2009 19923287 PMC2849269

[B41] Moya KL Benowitz LI Schneider GE Allinquant B The amyloid precursor protein is developmentally regulated and correlated with synaptogenesis Dev Biol 1994 2 597 603 10.1006/dbio.1994.1055S0012-1606(84)71055-4 10.1006/dbio.1994.1055 8314003

[B42] Sabo SL Ikin AF Buxbaum JD Greengard P The amyloid precursor protein and its regulatory protein, FE65, in growth cones and synapses in vitro and in vivo J Neurosci 2003 2 5407 5415 23/13/5407 12843239 10.1523/JNEUROSCI.23-13-05407.2003 PMC6741254

[B43] Storey E Beyreuther K Masters CL Alzheimer’s disease amyloid precursor protein on the surface of cortical neurons in primary culture co-localizes with adhesion patch components Brain Res 1996 2 217 231 0006-8993(96)00608-7 10.1016/0006-8993(96)00608-7 8911660

[B44] Yamazaki T Koo EH Selkoe DJ Cell surface amyloid beta-protein precursor colocalizes with beta 1 integrins at substrate contact sites in neural cells J Neurosci 1997 2 1004 1010 8994055 10.1523/JNEUROSCI.17-03-01004.1997 PMC6573178

[B45] Young-Pearse TL Chen AC Chang R Marquez C Selkoe DJ Secreted APP regulates the function of full-length APP in neurite outgrowth through interaction with integrin beta1 Neural Dev 2008 2 15 1749-8104-3-15 10.1186/1749-8104-3-15 10.1186/1749-8104-3-15 18573216 PMC2442059

[B46] Herms J Anliker B Heber S Ring S Fuhrmann M Kretzschmar H Sisodia S Müller U Cortical dysplasia resembling human type 2 lissencephaly in mice lacking all three APP family members Embo J 2004 2 4106 4115 10.1038/sj.emboj.7600390 15385965 PMC524337

[B47] Perez RG Zheng H Van der Ploeg LH Koo EH The beta-amyloid precursor protein of Alzheimer’s disease enhances neuron viability and modulates neuronal polarity J Neurosci 1997 2 9407 9414 9390996 10.1523/JNEUROSCI.17-24-09407.1997 PMC6573428

[B48] Allinquant B Hantraye P Mailleux P Moya K Bouillot C Prochiantz A Downregulation of amyloid precursor protein inhibits neurite outgrowth in vitro J Cell Biol 1995 2 919 927 10.1083/jcb.128.5.919 7876315 PMC2120404

[B49] Ando K Oishi M Takeda S Iijima K Isohara T Nairn AC Kirino Y Greengard P Suzuki T Role of phosphorylation of Alzheimer’s amyloid precursor protein during neuronal differentiation J Neurosci 1999 2 4421 4427 10341243 10.1523/JNEUROSCI.19-11-04421.1999 PMC6782598

[B50] Small DH Clarris HL Williamson TG Reed G Key B Mok SS Beyreuther K Masters CL Nurcombe V Neurite-outgrowth regulating functions of the amyloid protein precursor of Alzheimer’s disease J Alzheimers Dis 1999 2 275 285 12214125 10.3233/jad-1999-14-508

[B51] Leyssen M Ayaz D Hebert SS Reeve S De Strooper B Hassan BA Amyloid precursor protein promotes post-developmental neurite arborization in the Drosophila brain EMBO J 2005 2 2944 2955 7600757 10.1038/sj.emboj.7600757 10.1038/sj.emboj.7600757 16052209 PMC1187942

[B52] Ikin AF Sabo SL Lanier LM Buxbaum JD A macromolecular complex involving the amyloid precursor protein (APP) and the cytosolic adapter FE65 is a negative regulator of axon branching Mol Cell Neurosci 2007 2 57 63 S1044-7431(07)00028-0 10.1016/j.mcn.2007.02.003 10.1016/j.mcn.2007.02.003 17383198 PMC3622246

[B53] Gakhar-Koppole N Hundeshagen P Mandl C Weyer SW Allinquant B Müller U Ciccolini F Activity requires soluble amyloid precursor protein alpha to promote neurite outgrowth in neural stem cell-derived neurons via activation of the MAPK pathway Eur J Neurosci 2008 2 871 882 EJN6398 10.1111/j.1460-9568.2008.06398.x 10.1111/j.1460-9568.2008.06398.x 18717733

[B54] Hoe HS Lee KJ Carney RS Lee J Markova A Lee JY Howell BW Hyman BT Pak DT Bu G Rebeck GW Interaction of reelin with amyloid precursor protein promotes neurite outgrowth J Neurosci 2009 2 7459 7473 29/23/7459 10.1523/JNEUROSCI.4872-08.2009 10.1523/JNEUROSCI.4872-08.2009 19515914 PMC2759694

[B55] Deyts C Vetrivel KS Das S Shepherd YM Dupré DJ Thinakaran G Parent AT Novel GalphaS-protein signaling associated with membrane-tethered amyloid precursor protein intracellular domain J Neurosci 2012 2 1714 1729 32/5/1714 10.1523/JNEUROSCI.5433-11.2012 10.1523/JNEUROSCI.5433-11.2012 22302812 PMC3567462

[B56] Frotscher M Heimrich B Lamina-specific synaptic connections of hippocampal neurons in vitro J Neurobiol 1995 2 350 359 10.1002/neu.480260307 10.1002/neu.480260307 7775968

[B57] Frotscher M Zafirov S Heimrich B Development of identified neuronal types and of specific synaptic connections in slice cultures of rat hippocampus Prog Neurobiol 1995 2 vii xxviii 7598766 10.1016/0301-0082(94)00040-o

[B58] Gahwiler BH Capogna M Debanne D McKinney RA Thompson SM Organotypic slice cultures: a technique has come of age Trends Neurosci 1997 2 471 477 S0166-2236(97)01122-3 10.1016/S0166-2236(97)01122-3 9347615

[B59] Mallm JP Tschape JA Hick M Filippov MA Müller UC Generation of conditional null alleles for APP and APLP2 Genesis 2010 2 200 206 10.1002/dvg.20601 20140888 10.1002/dvg.20601

[B60] Shaked GM Chauv S Ubhi K Hansen LA Masliah E Interactions between the amyloid precursor protein C-terminal domain and G proteins mediate calcium dysregulation and amyloid beta toxicity in Alzheimer’s disease FEBS J 2009 2 2736 2751 EJB6997 10.1111/j.1742-4658.2009.06997.x 10.1111/j.1742-4658.2009.06997.x 19368557 PMC2838422

[B61] Sola Vigo F Kedikian G Heredia L Añel AD Rosa AL Lorenzo A Amyloid-beta precursor protein mediates neuronal toxicity of amyloid beta through Go protein activation Neurobiol Aging 2009 2 1379 1392 S0197-4580(07)00450-2 10.1016/j.neurobiolaging.2007.11.017 10.1016/j.neurobiolaging.2007.11.017 18187234

[B62] Wang B Yang L Wang Z Zheng H Amyolid precursor protein mediates presynaptic localization and activity of the high-affinity choline transporter Proc Natl Acad Sci U S A 2007 2 14140 14145 0704070104 10.1073/pnas.0704070104 10.1073/pnas.0704070104 17709753 PMC1955810

[B63] Tyan SH Shih AY Walsh JJ Maruyama H Sarsoza F Ku L Eggert S Hof PR Koo EH Dickstein DL Amyloid precursor protein (APP) regulates synaptic structure and function Mol Cell Neurosci 2012 2 43 52 10.1016/j.mcn.2012.07.009S1044-7431(12)00125-X 10.1016/j.mcn.2012.07.009 22884903 PMC3538857

[B64] Midthune B Tyan SH Walsh JJ Sarsoza F Eggert S Hof PR Dickstein DL Koo EH Deletion of the amyloid precursor-like protein 2 (APLP2) does not affect hippocampal neuron morphology or function Mol Cell Neurosci 2012 2 448 455 S1044-7431(12)00023-1 10.1016/j.mcn.2012.02.001 10.1016/j.mcn.2012.02.001 22353605 PMC3348437

[B65] Mucke L Abraham CR Masliah E Neurotrophic and neuroprotective effects of hAPP in transgenic mice Ann N Y Acad Sci 1996 2 82 88 10.1111/j.1749-6632.1996.tb34405.x 8624131

[B66] Bell KF Zheng L Fahrenholz F Cuello AC ADAM-10 over-expression increases cortical synaptogenesis Neurobiol Aging 2008 2 554 565 S0197-4580(06)00406-4 10.1016/j.neurobiolaging.2006.11.004 10.1016/j.neurobiolaging.2006.11.004 17187903

[B67] Marcello E Gardoni F Mauceri D Romorini S Jeromin A Epis R Borroni B Cattabeni F Sala C Padovani A Di Luca M Synapse-associated protein-97 mediates alpha-secretase ADAM10 trafficking and promotes its activity J Neurosci 2007 2 1682 1691 27/7/1682 10.1523/JNEUROSCI.3439-06.2007 10.1523/JNEUROSCI.3439-06.2007 17301176 PMC6673742

[B68] Hermann D Both M Ebert U Gross G Schoemaker H Draguhn A Wicke K Nimmrich V Synaptic transmission is impaired prior to plaque formation in amyloid precursor protein-overexpressing mice without altering behaviorally-correlated sharp wave-ripple complexes Neuroscience 2009 2 1081 1090 10.1016/j.neuroscience.2009.05.044S0306-4522(09)00956-7 10.1016/j.neuroscience.2009.05.044 19477243

[B69] Lannfelt L Basun H Wahlund LO Rowe BA Wagner SL Decreased alpha-secretase-cleaved amyloid precursor protein as a diagnostic marker for Alzheimer’s disease Nat Med 1995 2 829 832 10.1038/nm0895-829 7585189

[B70] Sennvik K Fastbom J Blomberg M Wahlund LO Winblad B Benedikz E Levels of alpha- and beta-secretase cleaved amyloid precursor protein in the cerebrospinal fluid of Alzheimer’s disease patients Neurosci Lett 2000 2 169 172 S0304394099009295 10.1016/S0304-3940(99)00929-5 10653020

[B71] Colciaghi F Borroni B Pastorino L Marcello E Zimmermann M Cattabeni F Padovani A Di Luca M [alpha]-Secretase ADAM10 as well as [alpha]APPs is reduced in platelets and CSF of Alzheimer disease patients Mol Med 2002 2 67 74 S1528365802200671 12080182 PMC2039975

[B72] Tyler SJ Dawbarn D Wilcock GK Allen SJ alpha- and beta-secretase: profound changes in Alzheimer’s disease Biochem Biophys Res Commun 2002 2 373 376 S0006291X02026359 10.1016/S0006-291X(02)02635-9 12445809

[B73] Fellgiebel A Kojro E Müller MJ Scheurich A Schmidt LG Fahrenholz F CSF APPs alpha and phosphorylated tau protein levels in mild cognitive impairment and dementia of Alzheimer’s type J Geriatr Psychiatry Neurol 2009 2 3 9 10.1177/08919887083278100891988708327810 10.1177/0891988708327810 19073834

[B74] Olsson A Hoglund K Sjogren M Andreasen N Minthon L Lannfelt L Buerger K Möller HJ Hampel H Davidsson P Blennow K Measurement of alpha- and beta-secretase cleaved amyloid precursor protein in cerebrospinal fluid from Alzheimer patients Exp Neurol 2003 2 74 80 S001448860300027X 10.1016/S0014-4886(03)00027-X 12957490

[B75] Anderson JJ Holtz G Baskin PP Wang R Mazzarelli L Wagner SL Menzaghi F Reduced cerebrospinal fluid levels of alpha-secretase-cleaved amyloid precursor protein in aged rats: correlation with spatial memory deficits Neuroscience 1999 2 1409 1420 S0306-4522(99)00244-4 10.1016/S0306-4522(99)00244-4 10501466

[B76] Obregon D Hou H Deng J Giunta B Tian J Darlington D Shahaduzzaman M Zhu Y Mori T Mattson MP Tan J Soluble amyloid precursor protein-alpha modulates beta-secretase activity and amyloid-beta generation Nat Commun 2012 2 777 10.1038/ncomms1781ncomms1781 22491325 10.1038/ncomms1781 PMC3520614

[B77] Bailey JA Ray B Greig NH Lahiri DK Rivastigmine lowers Abeta and increases sAPPalpha levels, which parallel elevated synaptic markers and metabolic activity in degenerating primary rat neurons PLoS One 2011 2 e21954 10.1371/journal.pone.0021954PONE-D-11-01100 10.1371/journal.pone.0021954 21799757 PMC3142110

[B78] Taylor CJ Ireland DR Ballagh I Bourne K Marechal NM Turner PR Bilkey DK Tate WP Abraham WC Endogenous secreted amyloid precursor protein-alpha regulates hippocampal NMDA receptor function, long-term potentiation and spatial memory Neurobiol Dis 2008 2 250 260 S0969-9961(08)00088-0 10.1016/j.nbd.2008.04.011 10.1016/j.nbd.2008.04.011 18585048

[B79] Claasen AM Guevremont D Mason-Parker SE Bourne K Tate WP Abraham WC Williams JM Secreted amyloid precursor protein-alpha upregulates synaptic protein synthesis by a protein kinase G-dependent mechanism Neurosci Lett 2009 2 92 96 S0304-3940(09)00664-8 10.1016/j.neulet.2009.05.040 10.1016/j.neulet.2009.05.040 19463893

