# Supplementary material for: Comparative analysis of single and combined APP/APLP knockouts reveals reduced spine density in APP-KO mice that is prevented by APPsα expression
Source: Acta Neuropathol Commun. 2014 Mar 31;2:36. doi: 10.1186/2051-5960-2-36 (PMC4023627; doi:10.1186/2051-5960-2-36)
Supplement: Additional file 1: Figure 1, Figure2, Figure 3 — Comparative analysis of single and combined APP/APLP knockouts reveals reduced spine density in APP-KO mice that is prevented by APPsα expression. [file 2051-5960-2-36-S1.pdf]

Additional File

Supplemental Figures 1-3

Comparative analysis of single and combined APP/APLP knockouts reveals reduced spine density in APP-KO mice that is prevented by APPsα expression

Sascha W. Weyer[1], Marta Zagrebelsky[3], Ulrike Herrmann [3], Meike Hick[1], Lennard Ganss[2], Julia Gobbert[1], Morna Gruber[4], Christine Altmann[4], Martin Korte[3], Thomas Deller[4], Ulrike C. Müller[1]

[1] Ruprecht-Karls University Heidelberg, Institute of Pharmacy and Molecular Biotechnology, Bioinformatics and Functional Genomics, Im Neuenheimer Feld 364, D-69120 Heidelberg, Germany

[2] Ruprecht-Karls University Heidelberg, Department of Applied Tumor Biology, Institute of Pathology, University of Heidelberg, and Clinical Cooperation Unit Applied Tumor Biology, German Cancer Research Center (DKFZ), D-69120 Heidelberg, Germany

[3] TU Braunschweig, Zoological Institute, Cellular Neurobiology, Spielmannstr. 7, D-38106 Braunschweig, Germany

[4] Goethe University Frankfurt, Institute of Clinical Neuroanatomy, Neuroscience Center, Theodor-Stern-Kai 7, D-60590 Frankfurt/Main

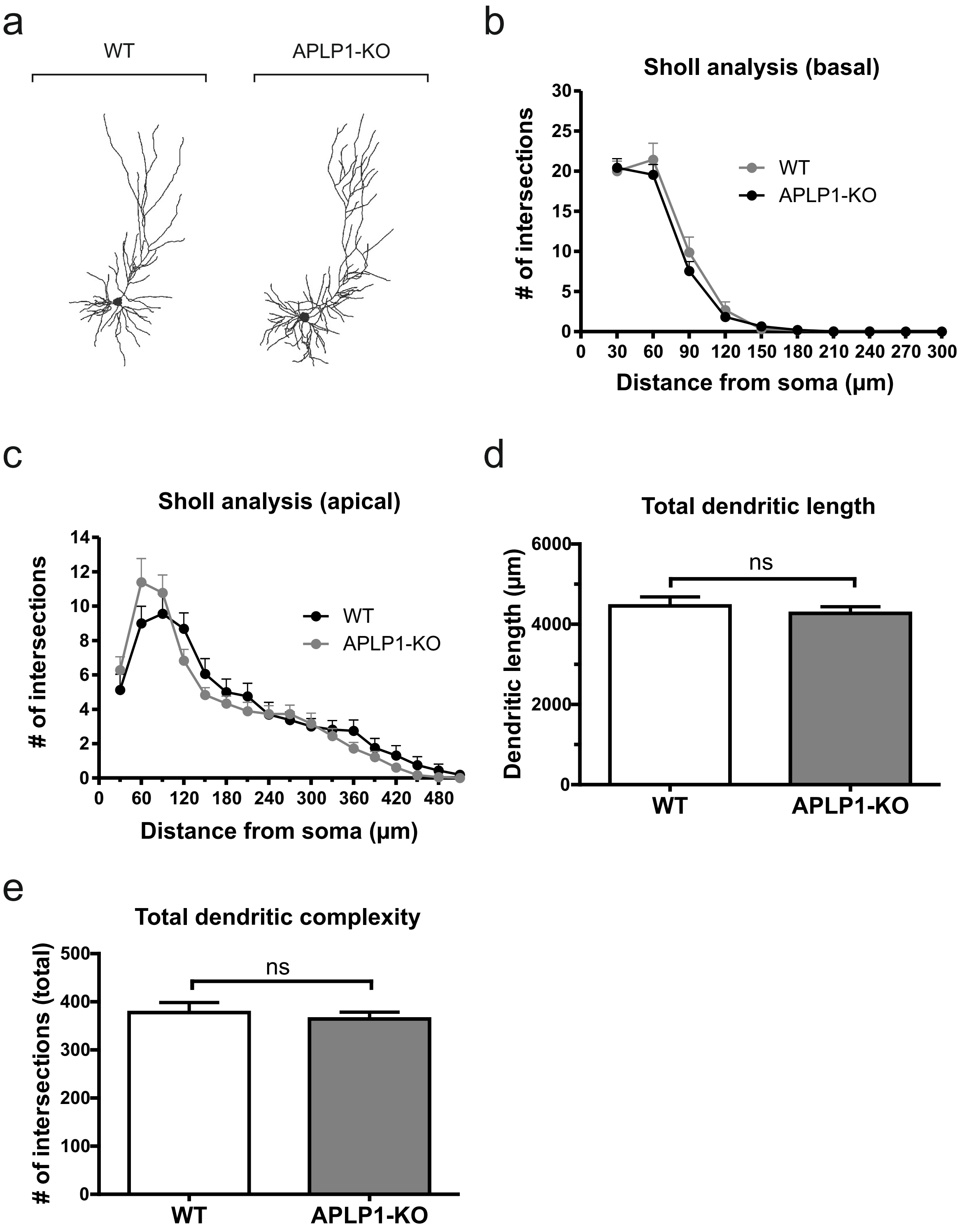

Suppl. Fig. 1

Suppl. Fig. 1: APLP1-KO does not lead to alterations in dendritic morphology.

(**a**) Representative examples of 3D-reconstructed hippocampal CA1 pyramidal neurons from WT and APLP1-KO OHCs. (**b, c**) Sholl analysis comparing basal (b) and apical (c) dendrites

between WT and APLP1-KO neurons revealed an unaltered branching pattern (Repeated measure ANOVA with Bonferroni multiple comparison test, n.s.). (**d**) Total dendritic length and (**e**) total dendritic complexity of APLP1-KO CA1 neurons was not significantly different from WT neurons (Student's t test, n.s.). WT: n = 22 neurons/ 5 mice, APLP1-KO: n = 27 neurons/ 6 mice). Values represent mean ± SEM.

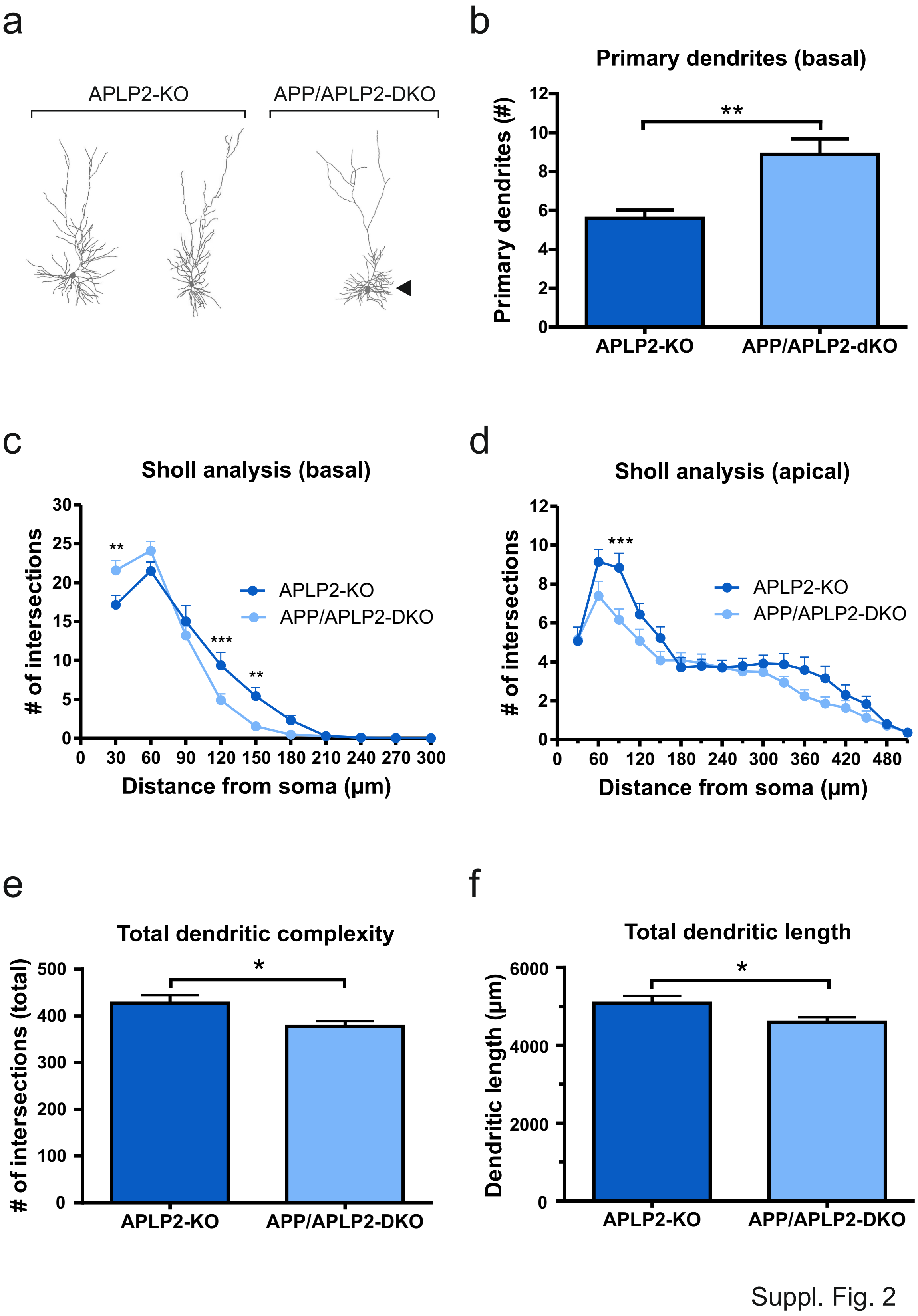

Suppl. Fig. 2: Pyramidal cells from combined APP/APLP2-DKO neurons show additional defects in dendritic complexity compared to APLP2-KO single mutants.

(**a**) Representative example of 3D-reconstructed CA1 neurons from APLP2-KO (left) and APP/APLP2-DKO mice (right). Arrowhead indicates an increase in the number of primary basal dendrites in APP/APLP2-DKO neurons. **(b**) Comparison of the number of primary basal dendrites revealed a striking increase in APP/APLP2-DKO neurons when compared to neurons from APLP2-KO littermate controls (Student´s t-test, $^{**}p \leq 0.01$). (**c**) Basal dendrites from APP/APLP2-DKO mice are characterized by an increase in complexity close to the soma (30 µm) and a decrease in distal aspects of basal dendrites (Repeated measure ANOVA with Bonferroni multiple comparison test: $^{**}p \leq 0.01$ for 30 µm and 150 µm, $^{***}p \leq 0.001$ for 120µm). (**d**) Sholl analysis of apical dendrites shows considerably decreased dendritic branching in proximal dendritic regions from APP/APLP2-DKO neurons compared to APLP2-KO neurons (Repeated measure ANOVA with Bonferroni multiple comparison test: $^{***}p \leq 0.001$ for 90 µm). Note that a similar reduction in dendritic complexity of double mutants was also seen in comparison to APP-KO single mutants (see Fig. 3). In mid-distal regions (330–390 µm), branching was reduced in APP/APLP2-DKO compared to APLP2-KO neurons, but failed to reach significance. APP/APLP2-DKO data in c, d same as in Fig. 3. (**e, f**) Alterations in neuronal morphology of APP/APLP2-DKO neurons resulted in a significant reduction in **(e)** the total dendritic complexity and **(f)** total dendritic length as compared to APLP2-KO littermate controls (Student´s t-test, $^{*}p \leq 0.05$). APLP2-KO: n = 25 neurons/ 5 mice, APP-DKO: n = 37 neurons/ 6 mice. All values represent mean ± SEM.

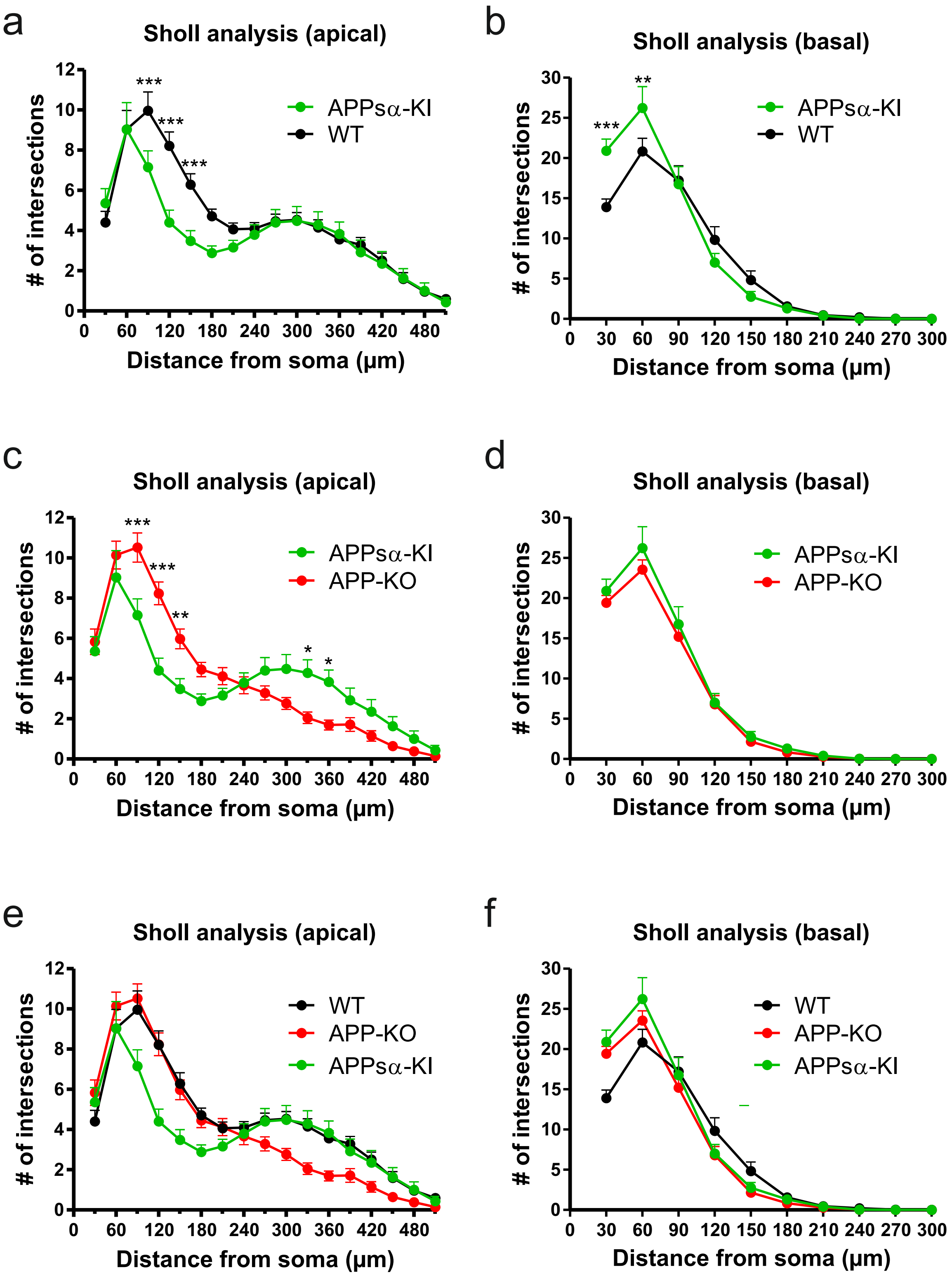

Suppl. Fig. 3

Suppl. Fig. 3: Sholl analysis of APPsα-KI neurons indicates a rescue in dendritic arborization restricted to distal segments of apical dendrites

(a) Compared to WT CA1 neurons apical dendrites of APPsα-KI neurons did not show the pronounced reduction of apical dendritic branching (at a distance of 300, 330, 360 μ from the soma) observed in APP-KO neurons (see Fig 2). At this distance from the soma Sholl graphs of WT and APPsα-KI neurons were indistinguishable (see also **e**), underscoring the fact that APPsα can rescue this *distal* dendritic phenotype. However, the role of APPsα for dendritic morphology appears more complex and dendritic branching was decreased in proximal apical dendrites. (Repeated measure ANOVA with Bonferroni´s multiple comparison test: ***p $\leq$ 0.001 for 120, 150 and 180μm). **(b)** Basal dendrites of APPsα-KI CA1 neurons revealed, similar to APP-KO neurons (see also **e**), an increase in the number of intersections close to the soma in (Repeated measure ANOVA with Bonferroni´s multiple comparison test: ***p $\leq$ 0.001 for 30μm; **p $\leq$ 0.01 for 60μm. (**c**) When compared to APP-KO neurons, APPsα-KI CA1 neurons show increased dendritic branching in distal segments of apical dendrites (Repeated measure ANOVA with Bonferroni´s multiple comparison test: *p $\leq$ 0.05 for 330 and 360μm). Reduced dendritic branching was observed in proximal segments of apical dendrites (Repeated measure ANOVA with Bonferroni´s multiple comparison test: ***p $\leq$ 0.001 for 120, 150μm, **p $\leq$ 0.01 for 180μm). **(d)** Branching pattern of basal dendrites showed no significant difference between APP-KO and APPsα-KI neurons. **(e, f)** Graphical superposition of Sholl graphs for WT, APP-KO and APPsα-KI neurons. Statistics were performed for two-group comparisons, as indicated above. **(a-c)** WT: n = 32 neurons/ 6 mice, APP-KO: n = 42 neurons/ 7 mice. APPsα-KI: n = 25 neurons/ 5 mice. All values represent mean ± SEM.
